# Transabdominal Robot-Assisted Laparoscopic Urethral Diverticulectomy of a Complex Anterior Horseshoe Diverticulum of the Proximal Urethra

**DOI:** 10.1089/cren.2015.29010.gsi

**Published:** 2015-10-01

**Authors:** Ganesh Sivarajan, Leonard Glickman, Kenneth Faber, Michelle Kim, Debra Fromer, Ravi Munver

**Affiliations:** Department of Urology, Hackensack University Medical Center, Hackensack, New Jersey.

## Abstract

Complex, proximal, anteriorly located urethral diverticula present the reconstructive urologist with a uniquely challenging task for repair through a conventional transvaginal approach. Herein, we present the first report of urethral diverticulectomy to excise a large, anterior, horseshoe-shaped urethral diverticulum that resulted in bladder outlet obstruction, using a transabdominal robot-assisted laparoscopic approach.

## Clinical History

**FIG. 1.**
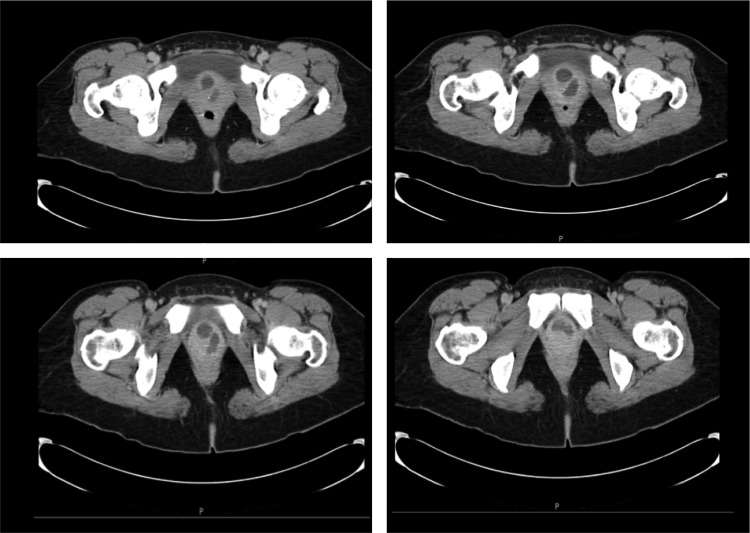
CT image of the large, proximal, and anterior urethral diverticulum that wraps around the urethra and bladder neck in a complex horseshoe configuration.

The patient is a 47-year-old female with a history of several weeks of worsening dysuria and urinary frequency, which persisted despite treatment of urine culture documented urinary-tract infections. She presented to the emergency department with a complaint of difficulty in initiating micturition and inability to empty her bladder. An abdominal ultrasound confirmed a diagnosis of acute urinary retention and identified a 3.4 cm lesion emanating from the region between the anterior vagina and the bladder. A CT scan revealed a complex cystic lesion at the base of the bladder, which appeared to originate from the urethra, consistent with an anteriorly located horseshoe-shaped urethral diverticulum (Fig. 1). She was discharged with a Foley catheter and was referred to a urologic specialist.

## Physical Examination

On physical examination, a urethral diverticulum could not be appreciated through vaginal examination. The contents of the diverticulum could not be expressed from the urethra due to its anterior and proximal urethral location. Cystourethroscopy revealed compression of the anterior bladder neck and a pinpoint ostium to the diverticulum at the left anterolateral aspect of the patient's proximal urethra, very close to the bladder neck. An MRI confirmed the presence of a 3.5 cm horseshoe-shaped urethral diverticulum encircling the proximal urethra anteriorly from the left across to the right side of the urethra, near the bladder neck.

## Diagnosis

The patient's history, cystoscopic findings, imaging results, and clinical scenario of recurrent UTIs and acute urinary retention were consistent with bladder outlet obstruction resulting from a large horseshoe-shaped urethral diverticulum. Urethral diverticula in females are a rare condition affecting <0.02% of women.^1^ These are generally repaired through a transvaginal approach either with or without a concurrent Martius interposition flap. In this patient, the diverticulum was not accessible transvaginally as it was located in the anterior aspect of the proximal urethra. The patient was scheduled to undergo transabdominal robot-assisted laparoscopic urethral diverticulectomy.

## Intervention

**FIG. 2.**
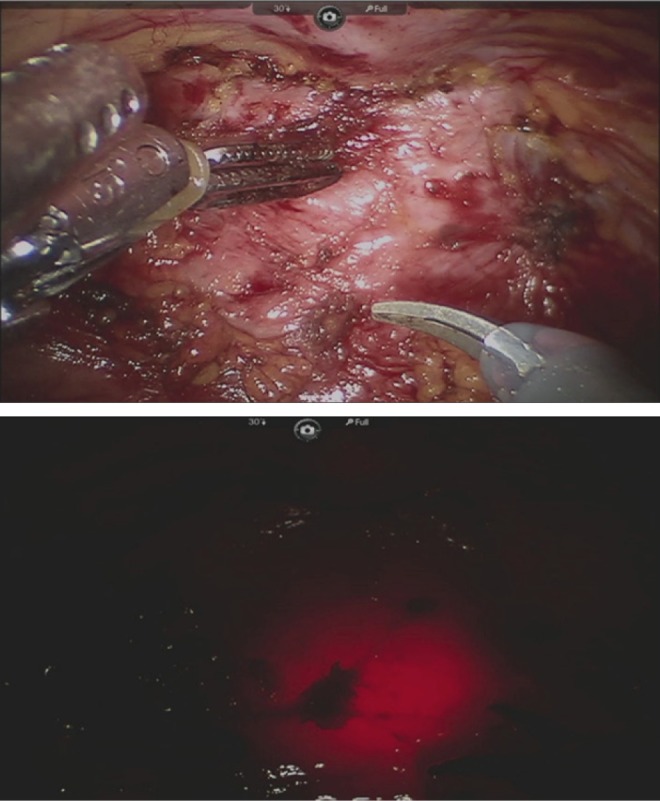
Precise identification of the region of the bladder neck despite the densely adherent and inflamed overlying diverticulum is facilitated by simultaneous cystoscopy with transillumination.

The patient was brought to the operating room and positioned in the lithotomy position. A vaginal examination was performed after induction of anesthesia, which confirmed that no portion of the diverticulum was palpable. The decision was made to proceed through a transabdominal laparoscopic approach using robotic assistance. Trocars were placed in the standard manner for transperitoneal robotic pelvic surgery using four trocars, including three robotic trocars and one assistant trocar. The robot was side-docked to allow transvanginal access to the urethra. The case was initiated by dividing the urachus and mobilizing the bladder off of the anterior abdominal wall to expose the retropubic space of Retzius. The endopelvic fascia was opened bilaterally exposing the region of the bladder neck and the proximal urethra. The anteriorly located diverticulum was decompressed and was difficult to identify as a result. Simultaneous transurethral cystoscopy with transillumination was utilized to identify the region of the bladder neck, which was obscured by the inflamed and adherent diverticulum (Fig. 2).

**FIG. 3.**
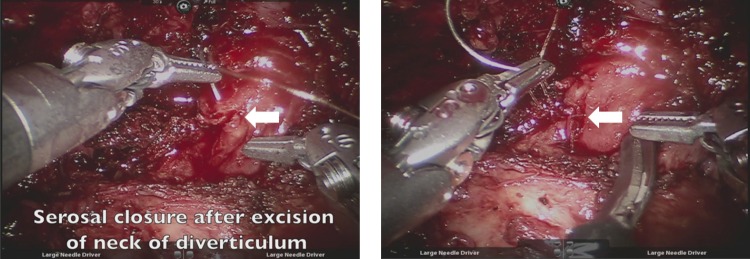
The neck of the diverticulum is transected, and the small serosal opening (*arrow*) in the urethra is closed with a single absorbable suture.

Transillumination through the cystoscope was also used to facilitate identification of the margins of the urethral diverticulum. The diverticulum was entered at its extreme left lateral margin to facilitate dissection without injury to the urethra. The wall of the diverticulum was carefully dissected off of the urethra until the neck of the diverticulum was identified. Once the neck of the diverticulum was transected, the resulting serosal entry was closed with an absorbable suture (Fig. 3). The remainder of the saddle diverticulum was then dissected from the urethra and submitted for pathologic assessment. Cystoscopy was then performed to confirm urethral patency and the absence of urethral injury. An 18F Foley catheter was placed and the patient was awakened from anesthesia without incident. The estimated blood loss was 20 mL, and the total operative time was 215 minutes.

## Follow-Up

The patient was discharged with a Foley catheter to gravity drainage on the second postoperative day. Final pathologic examination of the diverticulum revealed acute and chronic inflammation without evidence of malignancy. The patient underwent an effective voiding trial 1 week postoperatively.

## Outcome

At most recent follow-up 4 months post-operatively, the patient reported complete resolution of her preoperative voiding complaints and her postvoid residual was 0 mL. She had no symptoms or significant physical examination findings nor did she complain of any *de novo* postoperative voiding complaints, such as urinary frequency, urgency, dysuria, or incontinence.

This case represents a unique approach to the management of a large, proximal, horseshoe-shaped anterior urethral diverticulum causing bladder outlet obstruction, recurrent UTIs, and acute urinary retention. The majority of urethral diverticula can be managed through transvaginal excision with or without the use of an interposition graft (e.g., Martius) to help prevent postoperative fistula formation. While the rarity of this disease process makes the determination of treatment success rates difficult to ascertain, previous literature suggests that both proximal location and complex configuration (such as horseshoe-shape or circumferential) may be risk factors for treatment failure of transvaginal urethral diverticulectomy.^2,3^ For example, one series of 122 patients who underwent transvaginal excision of urethral diverticula found a 69.2% recurrence rate in the subset of patients with diverticula in the proximal urethra.^2^ Similarly, another series of transvaginal repairs reported no treatment failures among the 19 patients with simple diverticula but three treatment failures among the 17 patients with horseshoe-shaped or circumferential diverticuli.^3^

In fact, large, proximal anterior diverticula that extend into the retropubic space, particularly when in a complex horseshoe or circumferential configuration, are so challenging to effectively repair by a transvaginal approach that it has been suggested that the best outcomes are obtained with complete urethral transection followed by a formal urethroplasty either incorporating the anterior wall of the diverticulum, which must be left *in situ*, or in an end-to-end manner.^4^ In a small series of eight patients who underwent this technically challenging technique, postoperative complications such as urethrovaginal fistula and anastomotic stricture were reported.^4^

To our knowledge, this case represents the first report in the literature of a large, proximal, complex urethral diverticulum that was effectively managed through a transabdominal robotic approach. This novel approach offers several advantages over a transvaginal technique. This approach offers excellent exposure to an anteriorly located diverticulum that extends into the retropubic space. Robotic instrumentation can facilitate fine dissection of the diverticulum off of the adjacent urethra while avoiding the need for urethral transection and limiting inadvertent urethral entry, especially in the setting of inflammation and fibrosis that can result from previous infection. Moreover, the lack of overlapping suture lines within the vagina helps avoid the challenging complication of urethrovaginal fistula formation. Finally, this approach minimizes dissection along the pelvic floor and allows for precise identification and transection of the diverticular neck. Thus, only a small entry into the urethral serosa is created and can be easily closed with a single suture (Fig. 3). This obviates the need for complete urethral transection or formal urethral reconstruction, which may prevent the development of *de novo* voiding disorders postoperatively, such as incontinence or stricture formation.

In conclusion, we were able to treat a complex, proximal, anterior urethral diverticulum effectively through a robot-assisted laparoscopic transabdominal approach.

## Abbreviations Used

CT: computed tomography
MRI: magnetic resonance imaging
UTI: urinary-tract infection

## References

[1] El-Nashar SA, Bacon MM, Kim-Fine S, Weaver AL, Gebhart JB, Klingele CJ Incidence of female urethral diverticulum: A population-based analysis and literature review. Int Urogynecol J 2014;25:73–79 23857063 10.1007/s00192-013-2155-2 PMC4317296

[2] Ingber MS, Firoozi F, Vasavada SP, et al. Surgically corrected urethral diverticula: Long-term voiding dysfunction and reoperation rates. Urology 2011;77:65–69 20800882 10.1016/j.urology.2010.06.004

[3] Ockrim JL, Allen DJ, Shah PJ, Greenwell TJ A tertiary experience of urethral diverticulectomy: Diagnosis, imaging and surgical outcomes. BJU Int 2009;103:1550–1554 19191783 10.1111/j.1464-410X.2009.08348.x

[4] Rovner ES, Wein AJ Diagnosis and reconstruction of the dorsal or circumferential urethral diverticulum. J Urol 2003;170:82–86; discussion 86 12796650 10.1097/01.ju.0000067291.70172.b5

